# Unraveling the Skin Microbiome in Hidradenitis Suppurativa: Implications for Treatment and Disease Progression

**DOI:** 10.3390/jcm14072424

**Published:** 2025-04-02

**Authors:** Corina Ioana Cucu, Călin Giurcăneanu, Mara Madalina Mihai, Teodora Andronic, Ioan Ancuta, Mircea Ioan Popa, Ioana Sabina Macovei, Liliana Gabriela Popa

**Affiliations:** 1 Department of Dermatology, “Carol Davila” University of Medicine and Pharmacy, 020021 Bucharest, Romania; corina-ioana.cucu@drd.umfcd.ro (C.I.C.);; 2 Department of Dermatology, Colentina Clinical Hospital, 020125 Bucharest, Romania; 3 Department of Internal Medicine, “Carol Davila” University of Medicine and Pharmacy, 020021 Bucharest, Romania; 4 Department of Microbiology II, “Carol Davila” University of Medicine and Pharmacy, 020021 Bucharest, Romania

**Keywords:** hidradenitis suppurativa, skin microbiome, inflammation, antibiotic resistance

## Abstract

**Background:** Hidradenitis suppurativa (HS) is a chronic, disabling, and disfiguring inflammatory disease with a complex, incompletely elucidated pathogenesis. The role of skin dysbiosis in the development and progression of HS has not yet been clarified. **Methods:** We performed an observational, prospective culture-based study that included 40 HS patients and analyzed the bacterial load and diversity in HS skin lesions, their correlation with disease severity, and several host and environmental factors. Additionally, we investigated the prevalence of antibiotic resistance and determined the resistance profile of bacterial strains isolated from chronic HS lesions. **Results:** An impressive number and diversity of bacterial strains were isolated from both superficial and deep HS lesions. 201 aerobic and anaerobic bacterial strains were isolated, polymicrobial growth being detected in the majority of samples. The most frequently isolated bacteria were *Staphylococcus epidermidis*, *Staphylococcus aureus*, *Staphylococcus lugdunensis*, *Peptoniphilus* spp., and *Enterococcus faecalis* in superficial lesions and *Staphylococcus epidermidis*, *Staphylococcus aureus*, and *Corynebacterium tuberculostearicum* in deep lesions. A significantly higher bacterial density and diversity was found in male patients, regardless of the affected area and in patients with severe HS. The proportion of bacterial strains resistant to antibiotics was lower in our study (8.95%) compared to the previously reported data. **Conclusions:** Our findings indicate dysbiosis as a key player in the initiation and maintenance of the inflammatory process in HS. Further large-scale, prospective studies are required to comprehensively characterize the microbiological landscape of HS and shed light on its contribution in the pathogenesis of the disease.

## 1. Introduction

Hidradenitis Suppurativa (HS) is a chronic inflammatory disease of the pilosebaceous units, characterized by the development of painful inflammatory nodules, abscesses, sinus tracts, and fistulas draining purulent, malodorous discharge in intertriginous areas [1,2]. The inflammatory lesions tend to heal with deep and disfiguring scars, and permanently affect the skin’s appearance [3,4]. Additionally, contracture scars often lead to functional impairment. HS was first described by Velpeau in 1839 and defined by Verneuil in 1854 as a suppurative disease of the sweat glands, given the particular distribution of the lesions [3,4]. In 1992, following the differentiation between eccrine and apocrine sweat glands, Schiefferdecker further classified HS as a disorder of the apocrine glands [5]. One hundred years after the first report of HS, Brunsting assessed the histopathologic characteristics of the disease and concluded that the primary pathogenic processes in HS are follicular hyperkeratinization and follicular occlusion [6]. The prevalence of HS ranges from 0.053 to 4.1% [7,8]. Women are 2–5 times more often affected than men [9,10]. The mean age of onset is 23 [9], HS rarely occurring before the age of 11 and after the age of 50 [11,12]. The pathogenesis of HS is far from being elucidated, despite the great progress in unveiling the pathogenic pathways of the disease made in the past few decades [13,14,15]. Genetic susceptibility to an abnormal immune response and altered skin barrier function, hormonal imbalance, changes in the skin and gut microbiome, the superinfection of skin lesions with pathogenic bacteria, aberrant inflammatory and immune responses, autoinflammation, and environmental factors (obesity, smoking) are all involved in the development of HS [16,17,18,19]. Thus, the primum movens in HS development is represented by follicular occlusion [20,21]. This causes the progressive enlargement of the blocked follicle and eventually follicle rupture, inflammation that extends to the adjacent apocrine sweat glands and the formation of sinuous, interconnected draining tracts that become epithelialized. These offer optimal conditions for biofilm development, which, in turn, favors chronic inflammation, resistance to antibiotics, and impaired healing.

However, the role of bacteria in the development and progression of HS has not yet been clarified and continues to represent an attractive research field. Although, according to Koch’s postulates, HS is not an infectious disease in the classic meaning of the term, bacterial cultures from HS lesions frequently reveal polymorphic commensal flora [22] and the use of RNA metagenomics sequencing and prolonged culture methods has often led to the identification of pathogenic bacteria in HS lesions [23,24]. Some authors reported a positive correlation between the abundance of anaerobic bacteria and the presence of resistant bacterial species and HS severity [25]. Moreover, antibiotics have long been used in the treatment of HS, leading to temporary clinical improvement. Broader spectrum combination antibiotic therapy, like rifampin, moxifloxacin and metronidazole, and intravenous ertapenem, are associated with the rapid, significant amelioration of long-lasting severe HS lesions [26,27].

The aim of this study was to analyze the bacterial load and diversity in HS skin lesions and their correlation with the severity of the condition and to assess the impact of several factors, including the affected area, the severity of the disease, gender, and obesity on bacterial counts and diversity in HS. Another objective of the study was to investigate the prevalence of antibiotic resistance and to determine the resistance profile of bacterial strains isolated from chronic HS lesions. A better characterization of the bacterial population within HS lesions is an essential step in understanding the complex pathogenesis of the disease and enables a personalized therapeutic approach with enhanced efficacy.

## 2. Results

A total of 40 patients were included in the study, 29 (72.5%) of whom were men. The age of our patients ranged from 13 to 70 years, with a mean age of 32.9 ± 12.6 years.

A. Samples collected from superficial HS lesions

Samples from superficial HS lesions located in the axillary, inguinal, gluteal, and face and neck regions were collected in all 40 patients. A total of 120 bacterial strains belonging to 55 species were isolated, as depicted in Table 1. Polymicrobial growth (two or more species) was observed in the majority of bacterial cultures. The most frequently isolated bacteria were *Staphylococcus epidermidis* (11.6%), *Staphylococcus aureus* (8.3%), *Staphylococcus lugdunensis* (7.5%), *Peptoniphilus* spp. (5%), and *Enterococcus faecalis* (4.2%).

A notable difference between male and female patients was observed, with a significantly higher bacterial density in male patients for all strains, except for *Porphyromonas asaccharolytica*, *Streptococcus agalactiae*, and *Prevotella timonensis*, which were slightly more prevalent in female patients. A total of 92 (76.5%) of the bacterial strains were isolated from male patients.

1. Samples collected from superficial lesions located in the axillary region

In 19 patients, samples were collected from superficial HS lesions located in the axillary region. Of these patients, 14 (73.7%) were men. In eight samples (three obtained from female patients and five from male patients), only one bacterial strain was isolated, namely *Staphycococcus aureus*, *unspecified coagulase negative staphylococci*, *Staphylococcus epidermidis*, *Staphylococcus lugdunensis*, *Enterococcus faecalis*, and *Proteus mirabilis*. The rest of the bacterial cultures showed polymicrobial growth.

**Figure 1:**
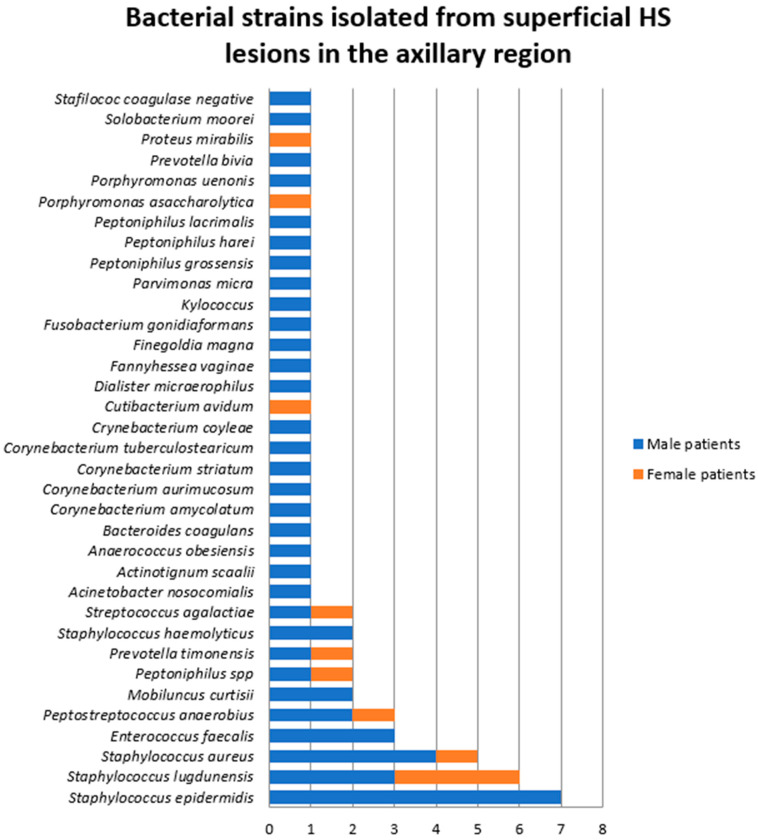
Bacterial strains isolated from superficial HS lesions in the axillary region.

A total of 59 bacterial strains belonging to 35 aerobic, anaerobic, facultative anaerobic, or microaerophilic species were identified in these specimens. The most frequent bacteria isolated from superficial axillary HS lesions was *Staphylococcus epidermidis* (11.8%), closely followed by *Staphylococcus lugdunensis* (10.2%), *Staphylococcus aureus* (8.5%), *Enterococcus faecalis* (5%), and *Peptostreptococcus anaerobius* (5%) (Figure 1).

The comparative analysis of bacterial samples isolated from male and female patients in superficial HS lesions located in the axillary region (Figure 1) showed a significantly higher bacterial count and diversity among male patients. *Cutibacterium avidum*, *Porphyromonas asaccharolytica*, and *Proteus mirabilis* were only found in female patients.

**Figure 2:**
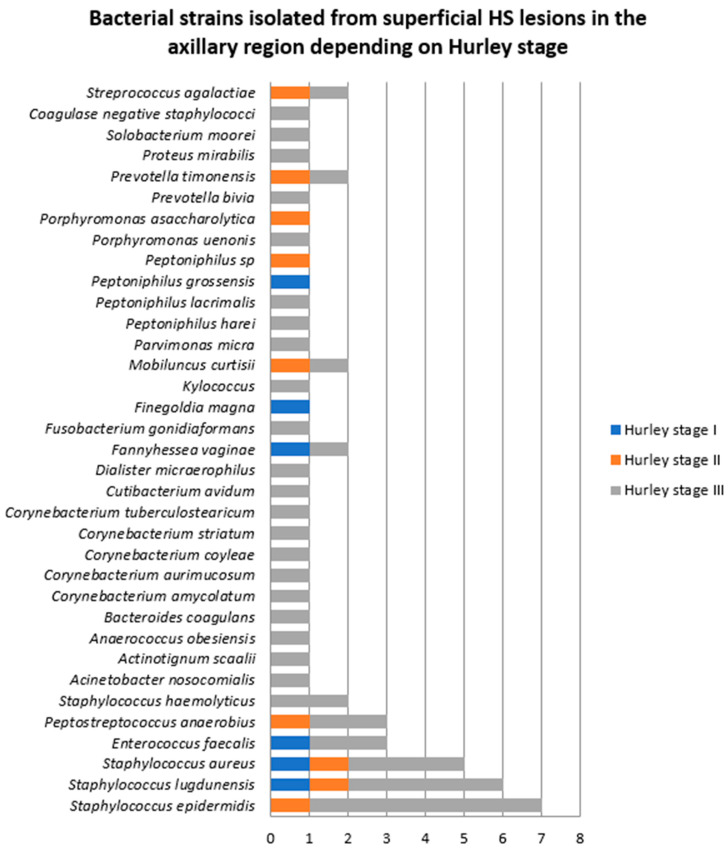
Bacterial strains isolated from superficial HS lesions in the axillary region depending on Hurley stage.

According to the Hurley severity staging system, three patients were diagnosed with HS Hurley stage I (two men and one woman), two patients with Hurley stage II (one man and one woman), and 14 patients with HS Hurley stage III (12 men and two women) [13]. Monobacterial growth was found in five patients with HS Hurley stage III, one patient with HS Hurley stage II and one patient with Hurley stage I. Polybacterial growth was detected in the rest of the samples. The majority of bacterial strains (44, 74.5%) were isolated from patients with HS Hurley stage III, followed by patients with HS Hurley stage II (9, 15.2%). *Staphylococcus epidermidis*, *Staphylococcus lugdunensis*, and *Staphylococcus aureus* were significantly more frequent in patients with HS Hurley stage III compared to patients with HS Hurley stage I and II (Figure 2).

**Figure 3:**
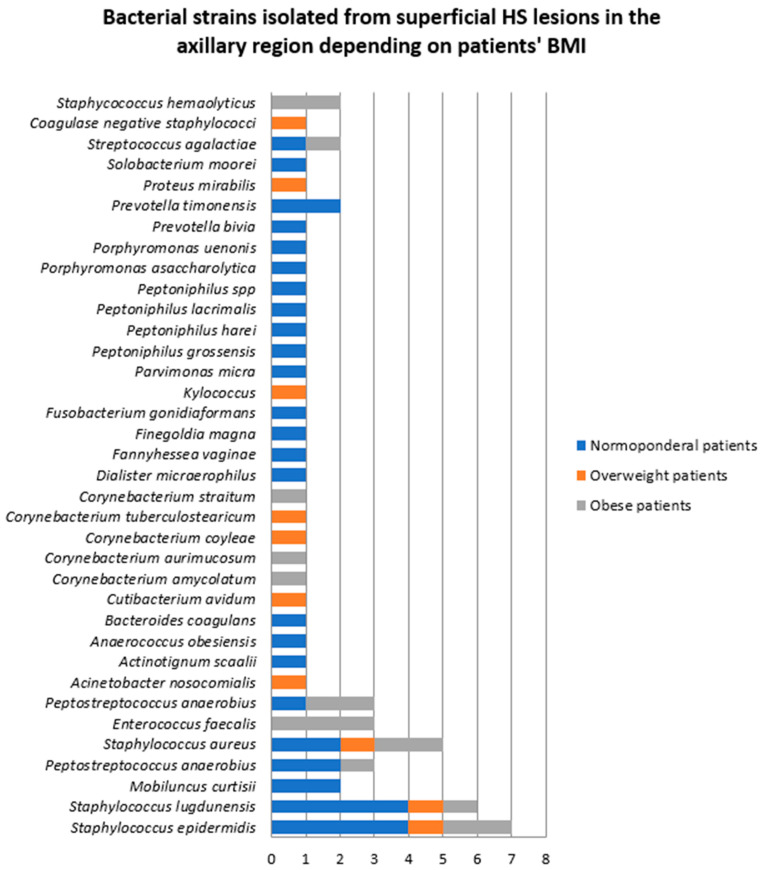
Bacterial strains isolated from superficial HS lesions in the axillary region depending on the patients’ body mass index (BMI).

The density and diversity of bacteria also varied significantly depending on the patients’ body weight (Figure 3). The number of bacterial strains isolated from superficial HS lesions located in the axillary region in normoponderal (eight patients), overweight (six patients), and obese patients (five patients) was 34 (57.6%), 10 (16.9%), and 15 (25.4%), respectively. The most frequently isolated bacteria from the axillary region in normoponderal patients were *Staphylococcus epidermidis* and *Staphylococcus lugdunensis*, whereas in obese patients, *Enterococcus faecalis*, *Staphylococcus epidermidis*, *Staphylococcus aureus*, and *Peptostreptococcus anaerobius* were most often identified.

2. Samples collected from superficial lesions located in the inguinal and gluteal regions

**Figure 4:**
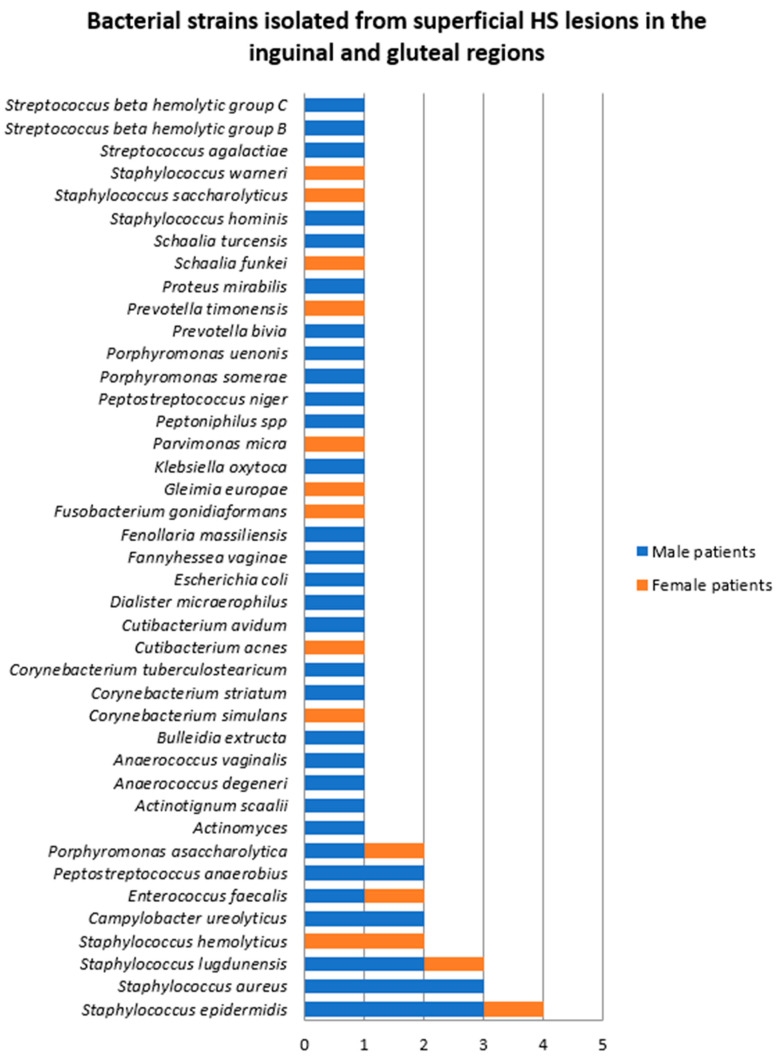
Bacterial strains isolated from superficial HS lesions in the inguinal and gluteal regions.

Samples were collected from superficial HS lesions located in the inguinal and gluteal regions in 16 patients, 12 (75%) of whom were men. A single bacterial strain was isolated from seven samples (one obtained from a female patient and six from male patients), namely *Staphycococcus aureus*, *Staphylococcus lugdunensis*, *Enterococcus faecalis*, *Escherichia coli*, and *Proteus mirabilis*. The rest of the cultures yielded polymicrobial results (Figure 4).

A total of 53 bacterial strains belonging to 41 species were identified. The most frequent bacteria isolated from inguinal and gluteal superficial HS lesions were *Staphylococcus epidermidis* (7.5%), *Staphylococcus aureus* (5.6%), and *Staphylococcus lugdunensis* (5.6%). Significantly higher bacterial density and diversity was found in samples collected from inguinal and gluteal superficial HS lesions of male patients.

**Figure 5:**
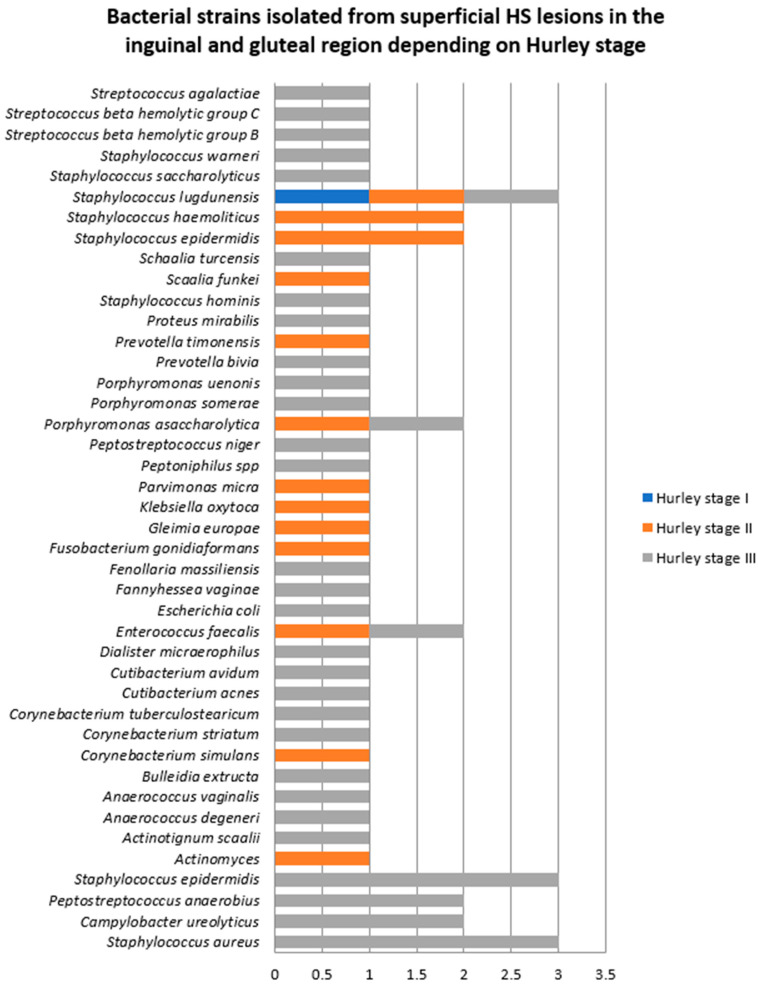
Bacterial strains isolated from superficial HS lesions in the inguinal and gluteal regions depending on Hurley severity stage.

Samples were collected from the inguinal and gluteal regions in one female patient with HS Hurley stage I, four patients with Hurley stage II (two men and two women), and 11 patients with Hurley stage III (10 men and one woman) (Figure 5). The majority of bacterial strains were isolated from patients with HS Hurley stage III (71.6%).

**Figure 6:**
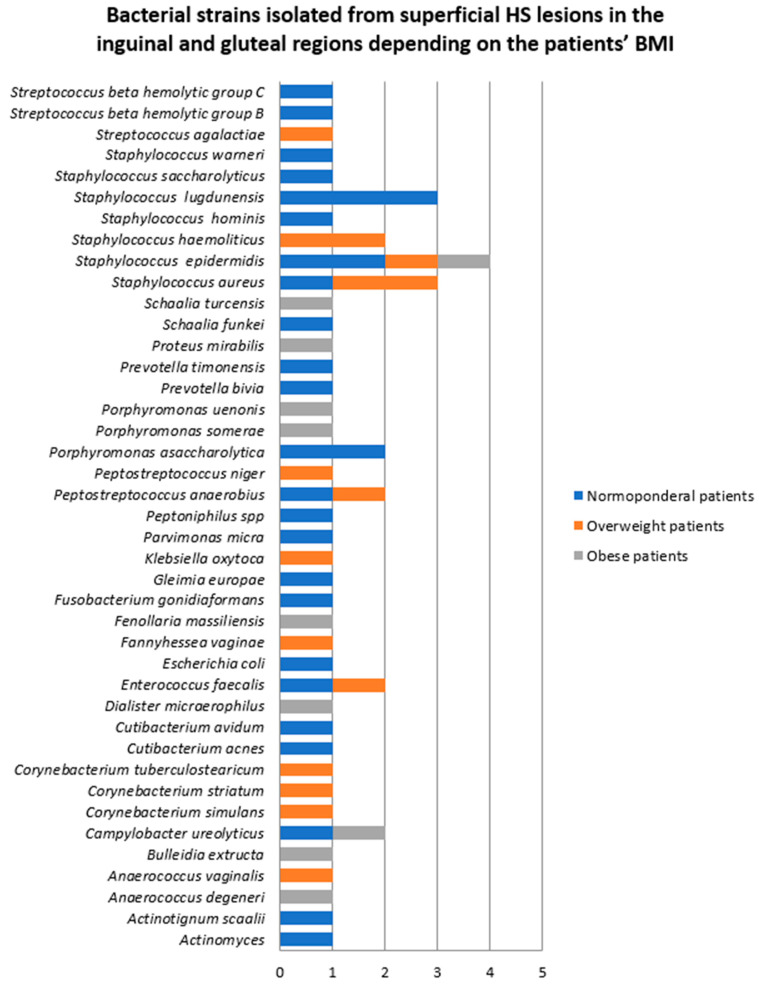
Bacterial strains isolated from superficial HS lesions in the inguinal and gluteal regions depending on the patients’ BMI.

The bacterial counts from samples obtained from the inguinal and gluteal regions also significantly differed depending on the patients’ BMI, as depicted in Figure 6. The number of bacterial strains isolated from normoponderal (11 patients), overweight (three patients), and obese (two patients) subjects were 28 (52.8%), 15 (28.3%), and 10 (18.5%), respectively. *Staphylococcus lugdunensis* was the most common bacteria in normoponderal patients, whereas in overweight patients *Staphylococcus aureus* was more common.

3. Samples collected from superficial lesions located in face and neck region

Samples were collected from superficial HS lesions located in the face and neck region in two female and two male patients. In three of the samples a single bacterial strain was detected, *Enterococcus faecalis*, *Staphylococcus epidermidis*, and coagulase negative staphylococci being isolated from these specimens.

**Figure 7:**
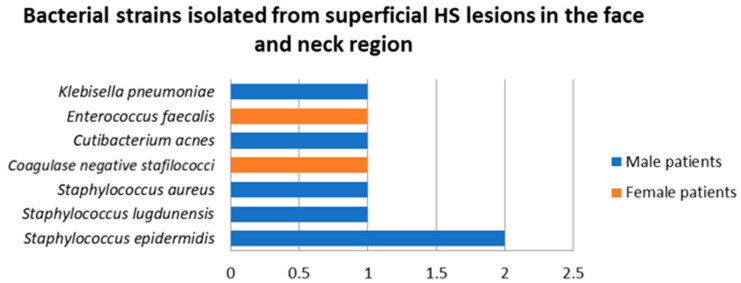
Bacterial strains isolated from superficial HS lesions in the face and neck region.

A total of eight bacterial strains belonging to seven species were isolated from superficial HS lesions in this area, the most common of which was *Staphylococcus epidermidis* (Figure 7). A significantly higher number of strains were isolated in male patients (6, 75%) compared to female patients (2, 25%).

**Figure 8:**
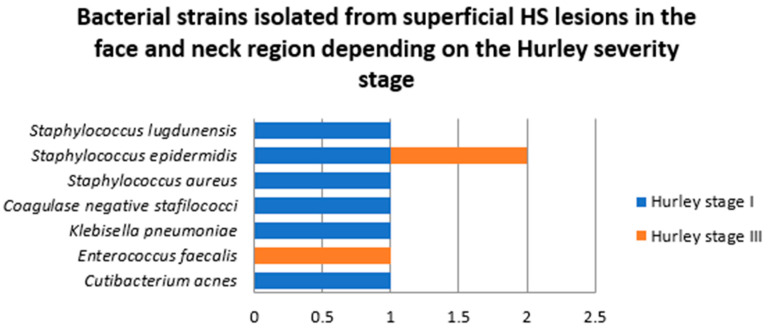
Bacterial strains isolated from superficial HS lesions in the face and neck region depending on the Hurley severity stage.

Two patients in this group were diagnosed with HS Hurley stage I (one female and one male patients) and two with HS Hurley stage III (one female and one male patients). A significantly higher number of strains was identified in samples collected from superficial face and neck HS lesions in patients with HS Hurley stage I (6, 75%) compared to HS Hurley stage III (2, 25%) (Figure 8).

**Figure 9:**
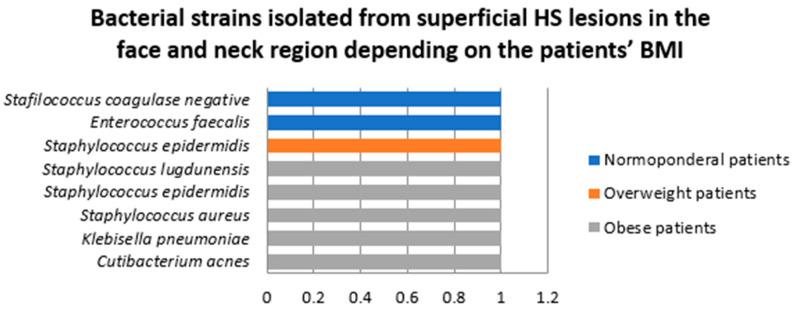
Bacterial strains isolated from superficial HS lesions in the face and neck region depending on the patients’ BMI.

Among patients in whom samples were collected from superficial HS lesions located in the face and neck region, two were normoponderal, one was overweight, and one was obese. A significantly higher number of bacterial strains were isolated from obese patients (five, 62.5%) compared to normponderal (two, 25%) and overweight patients (one, 12.5%) (Figure 9).

B. Samples collected from deep HS lesions

Samples were collected from deep HS lesions in 21 patients, of whom 17 (81%) were men. A total of 81 bacterial strains belonging to 42 species were isolated (Table 2). The most frequently isolated bacteria from deep HS lesions were *Staphylococcus epidermidis* (13.5%), followed by *Staphylococcus aureus* (8.6%), and *Corynebacterium tuberculostearicum* (7.4%). A significantly higher bacterial count and diversity was found in male patients. Notably, *Staphylococcus lugdunensis*, *Actinotignum schaalii*, *Campylobacter ureolyticus*, *Propionibacterium* spp., and *Actinobaculum massiliense* were only detected in samples collected from female patients.

1. Samples collected from deep HS lesions located in the axillary region

**Figure 10:**
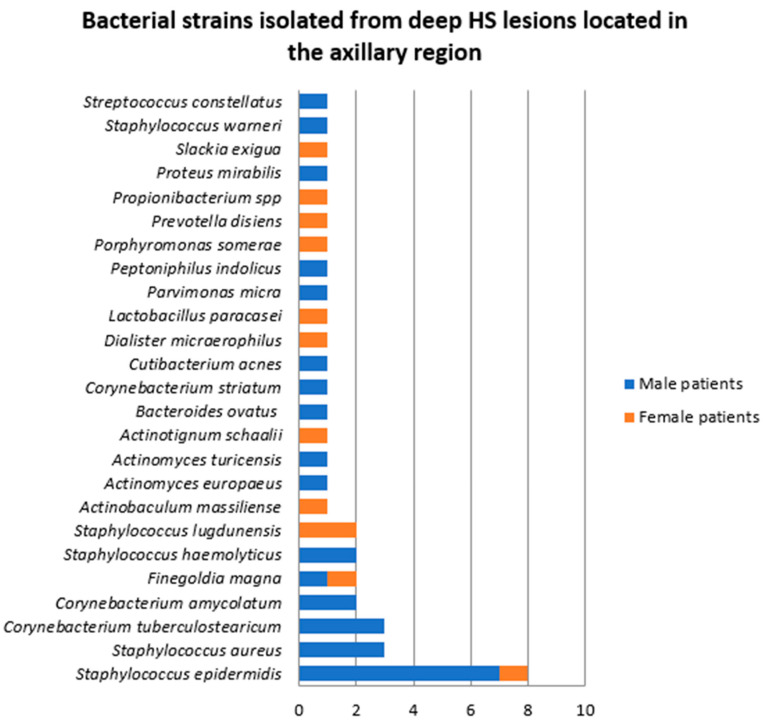
Bacterial strains isolated from deep HS lesions located in the axillary region.

In 10 patients (two women and eight men), the samples were collected from deep HS lesions located in the axillary region. In two of these samples, a single bacterial strain was identified, namely *Staphylococcus epidermidis*, and *Staphylococcus aureus*, respectively. The rest of the cultures yielded polymicrobial results. A total of 40 bacterial strains belonging to 25 species were identified, the most of common of which were *Staphylococcus epidermidis* (20%), *Staphylococcus aureus* (7.5%), and *Corynebacterium tuberculostearicum* (7.5%) (Figure 10). Male patients presented significantly higher bacterial density and diversity, 70% of the bacterial strains being detected in samples obtained from male patients.

**Figure 11:**
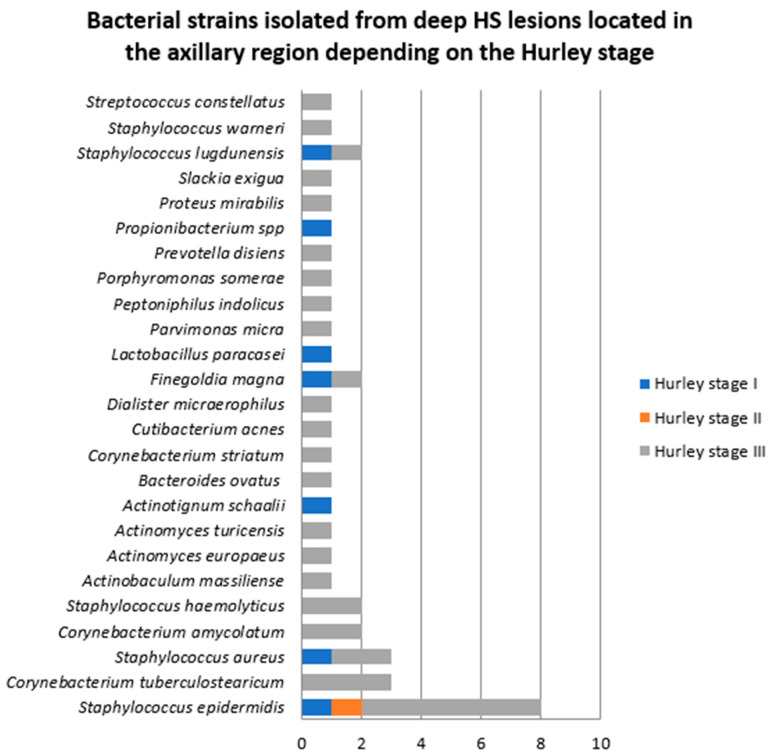
Bacterial strains isolated from deep HS lesions located in the axillary region depending on the Hurley stage.

Among the patients in whom bacterial samples were collected from deep axillary HS lesions, two were diagnosed with HS Hurley stage I (one female and one male patients), one male patient with HS Hurley stage II, and seven patients with HS Hurley stage III (one female and six male patients). The majority of bacterial strains were isolated from patients with HS Hurley stage III (32, 80%), as illustrated in Figure 11.

**Figure 12:**
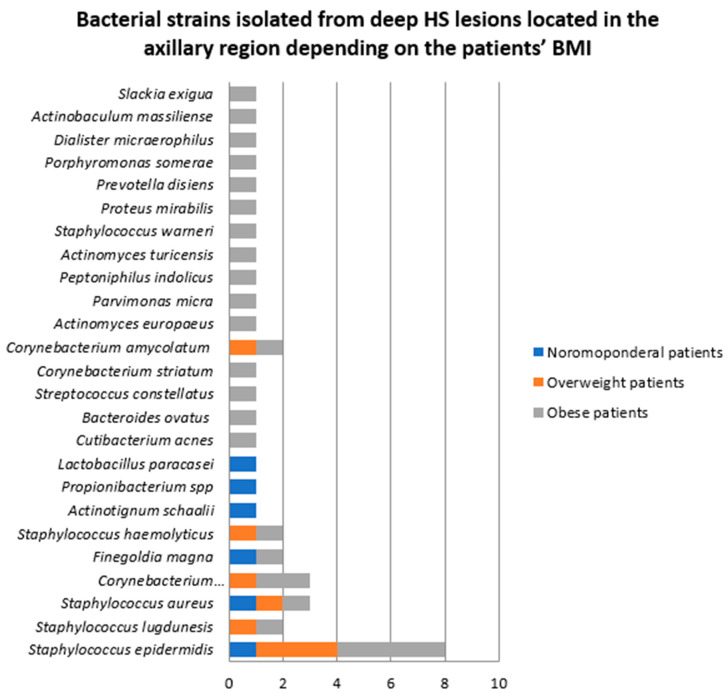
Bacterial strains isolated from deep HS lesions located in the axillary region depending on the patients’ BMI.

The number of bacterial strains isolated in patients with normal BMI (two patients), overweight (four patients) and obesity (four patients) was six (15%), eight (20%), and 26 (65%), respectively (Figure 12).

2. Samples collected from deep HS lesions located in the inguinal and gluteal regions

**Figure 13:**
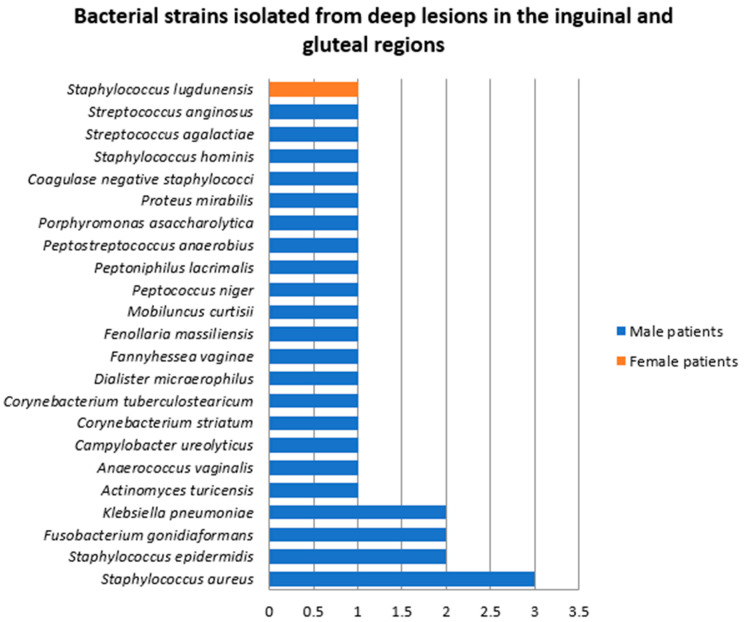
Bacterial strains isolated from deep HS lesions located in the inguinal and gluteal regions.

Samples were collected from deep HS lesions located in the inguinal and gluteal areas in nine patients, of whom eight (89%) were men. In five of the samples, a single strain was found (*Staphylococcus aureus*, *Staphylococcus epidermidis*, *Staphylococcus lugdunensis*, unspecified coagulase negative staphylococcus, and *Klebsiella pneumoniae*). Polymicrobial growth was found in four samples (Figure 13). A total of 28 bacterial strains belonging to 23 species were identified. The most frequent bacteria isolated from deep HS lesions located in the inguinal and gluteal regions were *Staphylococcus aureus* (10.7%), *Staphylococcus epidermidis* (7.1%), *Fusobacterium gonidiaformans* (7.1%), and *Klebsiella pneumoniae* (7.1%).

**Figure 14:**
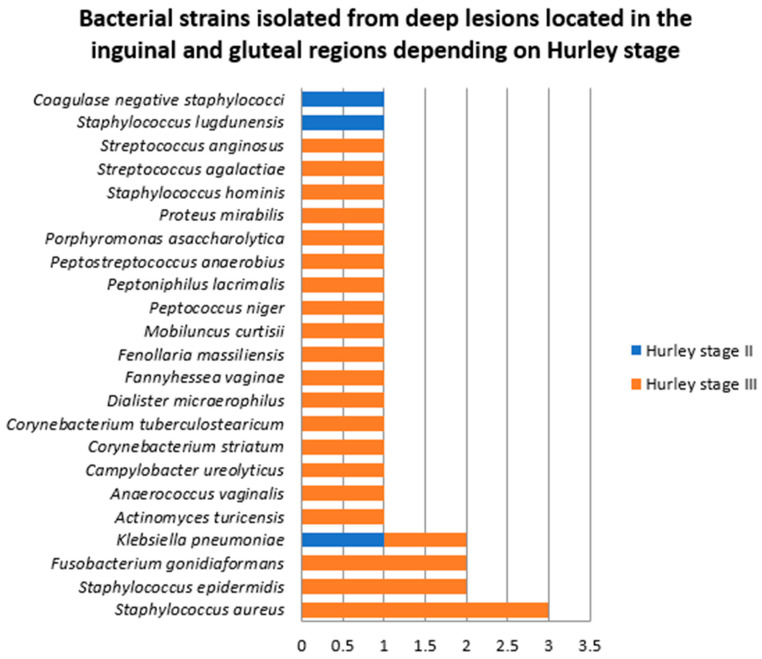
Bacterial strains isolated from deep lesions located in the inguinal and gluteal regions depending on Hurley stage.

Patients from whom samples were collected from deep HS lesions located in the inguinal and gluteal lesions were diagnosed with HS Hurley stage II (two patients) and HS Hurley stage III (seven patients). The differences in the number and diversity of bacterial strains between patients with HS Hurley stage II and III are presented in Figure 14. Most of the bacterial strains were isolated in patients with HS Hurley stage III (25, 90%).

**Figure 15:**
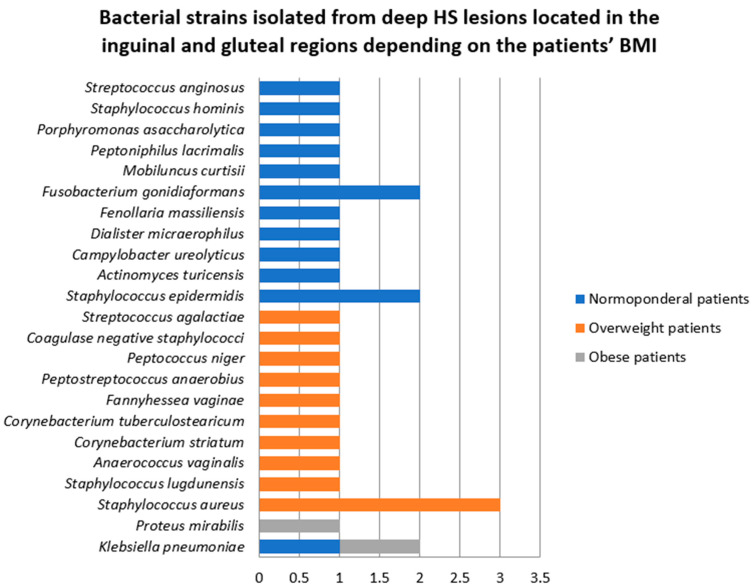
Bacterial strains isolated from deep HS lesions located in the inguinal and gluteal regions depending on the patients’ BMI.

The majority of bacterial strains were detected in the four normoponderal patients (14, 50%), closely followed by the four overweight patients (12, 42.8%). In the only obese patient in whom samples were prelevated from deep inguinal lesions, two strains (*Klebsiella pneumoniae* and *Proteus mirabilis*) were isolated (Figure 15).

3. Samples collected from deep HS lesions in the face and neck region

In two patients, both normoponderal females, samples were collected from deep face and neck HS lesions. In one patient, diagnosed with HS Hurley stage I, coagulase negative staphylococcus was detected. In the other patient, diagnosed with HS Hurley stage III, the bacterial cultures yielded polymicrobial results, *Campylobacter ureolyticus* and *Enterococcus faecalis* being isolated.

C. Antibiotic resistance profile of bacterial strains isolated from HS lesions

Of the total 201 bacterial strains isolated in our study group from superficial and deep HS lesions, 18 (8.95%) exhibited antibiotic resistance, as depicted in Table 3. *Staphylococcus aureus* was the most frequent bacteria resistant to one or multiple antibiotics. Notably, one methicillin-resistant *Staphylococcus aureus* strain (MRSA) and one *Staphylococcus aureus* strain displaying both methicillin resistance and inducible clindamycin resistance (MLSBi) were identified.

## 3. Discussion

Considering the complex interaction and bidirectional influence between the cutaneous flora and the innate and adaptive immune system, the changes in the skin microbiome and its dysregulated interaction with the host in HS have been investigated [28,29,30,31,32,33,34,35,36,37,38,39,40,41,42]. In our study, an impressive number and diversity of bacterial strains were isolated from both superficial and deep HS lesions. Bacterial cultures led to the identification of 120 bacterial strains belonging to 55 species in samples collected from superficial HS lesions in all 40 patients included in our study and of 81 bacterial strains belonging to 42 species in samples collected from deep HS lesions in 21 of our patients. Polymicrobial growth was detected in the majority of the samples. In accordance with previously reported data, the most frequently isolated bacteria in superficial HS lesions were *Staphylococcus epidermidis*, *Staphylococcus aureus*, *Staphylococcus lugdunensis*, *Peptoniphilus* spp., and *Enterococcus faecalis*. The most common bacteria identified in deep HS lesions were *Staphylococcus epidermidis*, followed by *Staphylococcus aureus*, and *Corynebacterium tuberculostearicum*.

A considerable difference between male and female patients was observed, with a significantly higher bacterial density and diversity in male patients in both superficial and deep HS lesions, regardless of the sampled area.

Notable differences were also detected depending on the affected area. The most frequent bacteria isolated from superficial axillary lesions were *Staphylococcus epidermidis, Staphylococcus lugdunensis*, *Staphylococcus aureus*, *Enterococcus faecalis*, and *Peptostreptococcus anaerobius*, whereas in deep axillary lesions, *Staphylococcus epidermidis*, *Staphylococcus aureus*, and *Corynebacterium tuberculostearicum* were most often detected. Similar results were obtained from the inguinal and gluteal superficial lesions, *Staphylococcus epidermidis*, *Staphylococcus aureus*, and *Staphylococcus lugdunensis* being the most commonly encountered bacteria. However, the most frequent bacteria isolated from deep inguinal and gluteal HS lesions differed from those isolated from deep axillary lesions, being represented by *Staphylococcus aureus*, *Staphylococcus epidermidis*, *Fusobacterium gonidiaformans*, and *Klebsiella pneumoniae*. Superficial face and neck HS lesions were most commonly colonized with *Staphylococcus epidermidis*, whereas coagulase negative staphylococci, *Campylobacter ureolyticus and Enterococcus faecalis* were detected in deep face and neck HS lesions.

Unsurprisingly, the samples collected from patients with HS Hurley stage III showed a significantly higher bacterial count and diversity compared to samples obtained from patients with milder disease regardless of the affected area.

Contrary to our expectations, a significantly higher number of bacterial strains were isolated from superficial axillary, inguinal and gluteal HS lesions in normoponderal patients compared to overweight and obese patients. The opposite was observed for superficial HS lesions located in the head and neck region. The most frequently isolated bacteria from superficial HS lesions located in the axillary, inguinal and gluteal regions in normoponderal patients were *Staphylococcus epidermidis* and *Staphylococcus lugdunensis*, whereas in obese patients, *Enterococcus faecalis*, *Staphylococcus epidermidis*, *Staphylococcus aureus*, and *Peptostreptococcus anaerobius* were most often identified. Conversely, the number of bacterial strains isolated from deep axillary HS lesions was significantly greater in obese patients compared to overweight and normoponderal subjects.

Our study’s findings align with the existing literature regarding the diversity of bacterial species isolated from HS lesions. However, comparing conclusions across published studies can be challenging due to variations in sample collection and microbiological analysis methods. Nonetheless, the key findings of our analysis support the previously established diversity, with multiple commensal species detected in these lesions [43,44].

In our analysis, *Staphylococcus aureus* was the most prevalent species isolated superficially and deeply. Multiple studies investigated the role of *Staphylococcus aureus* in the early stages of the disease, suggesting its temporary involvement [45]. Using carbon dioxide laser surgery, Lapins et al. obtained samples from various depths of the lesions and identified 16 different bacterial species [46]. In all cases, bacterial cultures were positive at least at one level, and in all but three cases, they were also positive at deeper levels. *Staphylococcus aureus* and *coagulase-negative Staphylococci* were the most frequently isolated species [38,47,48,49,50,51]. Attempts have been made to correlate it with disease stage, however, the conclusions have been inconsistent. Consequently, the specific role of this species remains unclear, despite being one of the most frequently isolated.

*Staphylococcus epidermidis*, a member of the coagulase-negative staphylococci, is commonly regarded as a commensal organism, which has the potential to cause significant infections in immunosuppressed individuals [52]. In their review, Ring et al. highlighted the considerable presence of this species in the analyzed samples [45]. They raised the question of whether a potential commensal could play a role in the inflammatory process of HS, while also emphasizing the possibility of accidental contamination with a commensal during sample collection [45]. Another member of this group, *Staphylococcus lugdunensis*, was frequently identified in our study. It has previously been recognized as an uniquely virulent species, most commonly affecting the pelvic region and lower limbs and causing abscesses similar to those produced by Staphylococcus aureus [53]. A study conducted in France identified *Staphylococcus lugdunensis* as a predominant species in HS lesions and correlated its presence with Hurley stage I. This finding led the authors to hypothesize that it may contribute to skin inflammation in the early phase of the condition [23].

Among the diverse species we have identified, it is worth noting that a considerable proportion are anaerobic and facultative anaerobic species, including *Enterococcus faecalis*, *Actinomycetes*, as well as other species that are normally found in commensal cutaneous and gastrointestinal environments [54]. Anaerobic bacteria have been identified as causative agents of skin abscesses in various clinical contexts and have been isolated in multiple studies. In their meta-analyses of 22 studies, Williams et al. suggested that anaerobic Gram-negative bacteria, particularly the genera *Porphyromonas*, *Prevotella*, and *Bacteroides*, exhibit the greatest potential involvement in the pathogenesis of HS, as evidenced by their frequent presence in HS lesions [55]. Their ability to proliferate may be attributed to cutaneous dysbiosis, partly explained by the reduced number of sebaceous glands in HS. This reduction leads to a decrease in *Cutibacterium* spp. within HS lesions, thereby promoting the formation of biofilms by other commensal bacteria and enhancing their pathogenic potential [56,57,58,59,60,61,62,63,64].

The response to anti-inflammatory treatments, including biologics, is highly variable among HS patients, once again highlighting the complexity of the disease. Antibiotics, especially products with intrinsic anti-inflammatory and immunomodulatory properties are widely used for the control of HS. Nevertheless, antibiotic resistance is increasingly frequent and complicates treatment even further. Reported resistance rates in HS patients are as high as 55–65.7% [39,65] for clindamycin, 69.3% for rifampicin [65], and exceed 70% for penicillin, ciprofloxacin, tetracycline, and erythromycin used as monotherapy [65]. In our study, antibiotic resistance rate was much lower, found in 18 (8.95%) bacterial strains. Resistance to antibiotics was especially detected in *Staphylococcus aureus* strains, one methicillin-resistant *Staphylococcus aureus* strain and one *Staphylococcus aureus* strain displaying both methicillin resistance and inducible clindamycin resistance having been identified.

## 4. Materials and Methods

We performed an observational cohort study in which 40 patients diagnosed with chronic HS were enrolled over 4 years. Informed consent was obtained from each participant before study initiation. The study adhered to the ethical principles outlined in the 1975 Declaration of Helsinki and Good Clinical Practice (GCP) standards and received approval from the Ethics Commission of our institution.

The study included females and males aged 18 to 70 years, diagnosed with HS who presented active inflammatory lesions in at least one anatomical region. The subjects must have had HS skin lesions for at least 1 year. Patients from all Hurley stages were included in the study. Patients with other active diseases that could interfere with bacteriological investigations, such as cutaneous and systemic infections, were excluded from our study. Patients who had used topical antibiotic treatments (except local antiseptics or antibacterial body washes) within 30 days prior to sample collection or had received systemic antibiotic treatment in the month before sample collection were also not eligible for participation.

The diagnosis of HS was established based on the presence of typical lesions, including inflammatory nodules, comedones, and fistulous tracts located in intertriginous areas, with a chronic course of more than three months. Patients were classified into three severity stages according to Hurley classification [13]. A general physical examination was performed, and standardized clinical photographs were taken. Patients with a body mass index (BMI) of 18.5–24.9 were classified as normoponderal, patients with BMI of 25–29.9 as overweight, and patients with a BMI exceeding 30 as obese. A thorough patient interview was conducted to obtain data regarding the family history of HS or other relevant disorders, age of the patient at the onset of the disease, the lesions’ type and distribution throughout the course of the disease, comorbidities, body mass index, previous treatments and the disease response, and quality of life assessments.

Samples were collected from both superficial and deep HS sites. The lesions (inflammatory nodules on the skin, superficial fistulas, draining abscesses, etc.) were selected based on the level of discomfort experienced by the patient, and factors such as inflammation, pain, recurrence, and suppuration. The most recent and active lesions were chosen for bacterial sampling [66]. First lesion—using sterile swabs without prior skin asepsis, samples were collected from superficial purulent HS lesions and immediately placed in a rapid transport buffer for prompt plating on various culture media, including Thioglycollate Broth with Resazurin THIO-T (bioMérieux). Second lesion—for deep, closed HS lesions, that had no visible connection to the surface of the skin, tegument asepsis using 10% povidone-iodine solution was performed. After that, deep lesions were peterated using a disposable punch biopsy device, and the purulent discharge was collected with a sterile swab. Microbial cultures were processed using both aerobic and anaerobic techniques. Gram staining was performed on all specimens, and pure colonies were isolated by inoculating each swab onto 5% sheep blood agar, Chocolate agar, MacConkey agar (without crystal violet), and Sabouraud agar with chloramphenicol (Oxoid). All plates, except those on Sabouraud agar, were incubated aerobically at 37 °C for 18–24 h, while Sabouraud plates were incubated at both 30 °C and 37 °C for 24–72 h. Biochemical identification of bacterial strains was conducted using the automated Vitek 2 system (bioMérieux) and Phoenix BD (Becton–Dickinson). Following identification, bacterial strains were stored at 4 °C. For further experiments, strains were streaked onto nutrient agar and incubated overnight at 37 °C.

## 5. Limitations

While this study provides valuable insights, several limitations must be acknowledged, highlighting the need for further research. Firstly, the relatively small sample size may restrict the applicability of our findings to the broader HS population. Additionally, using swabs to sample superficial lesions could be replaced with needle aspiration to minimize contamination from commensal flora. Furthermore, bacterial identification was based solely on culture-dependent methods, potentially overlooking uncultivable or fastidious microorganisms. Future research involving larger cohorts and advanced metagenomic techniques is warranted to achieve a more comprehensive understanding of microbial dynamics in HS and to allow the development of more effective therapeutic strategies.

## 6. Conclusions

A diverse range of aerobic and anaerobic bacteria was isolated from the HS lesions in our patients. Consistent with previous studies, coagulase negative staphylococci and *Staphylococcus aureus* were the most frequently identified species, regardless of the location of the skin lesions. Our findings support previously proposed hypotheses, according to which the commensal skin flora, though still not fully characterized, plays a key role in the initiation and maintenance of the inflammatory process in HS. The presence of various bacterial species in both superficial and deep HS lesions, in relatively similar proportions, highlights a continuum of microbial involvement, independent of lesion depth.

Our study reinforces existing evidence on the role of secondary bacterial infection in HS pathogenesis and its contribution to the persistence of chronic suppurative lesions. However, identifying the specific bacterial groups that play a definitive role in disease progression remains challenging. Given the anti-inflammatory and antimicrobial effects of antibiotic therapy in HS, optimized regimens can significantly improve disease severity, particularly in severe cases. To balance the benefits and risks of prolonged antimicrobial treatment and better understand bacterial susceptibility patterns, future large-scale prospective studies should incorporate antibiogram analysis to refine personalized antibiotic strategies and further elucidate the microbiological landscape of HS.

## Figures and Tables

**Table 1:** Bacterial strains isolated from superficial HS lesions.

Bacterial Strains Isolated from Superficial HS Lesions	Total	Female Patients	Male Patients
*Staphylococcus epidermidis*	14	1	13
*Staphylococcus aureus*	10	1	9
*Staphylococcus lugdunensis*	9	4	5
*Peptoniphilus spp*	6	1	5
*Enterococcus faecalis*	5	2	3
*Proteus mirabilis*	4	1	3
*Staphylococcus haemolyticus*	4	2	2
*Porphyromonas asaccharolytica*	3	2	1
*Streptococcus agalactiae*	3	2	1
*Actinotignum scaalii*	2	0	2
*Campylobacter ureolyticus*	2	0	2
*Cutibacterium acnes*	2	1	1
*Cutibacterium avidum*	2	1	1
*Dialister micraerophilus*	2	0	2
*Fannyhessea vaginae*	2	0	2
*Fusobacterium gonidiaformans*	2	1	1
*Mobiluncus curtisii*	2	0	2
*Parvimonas micra*	2	1	1
*Porphyromonas uenonis*	2	0	2
*Prevotella bivia*	2	0	2
*Prevotella timonensis*	2	2	0
*Coagulase negative staphilococci*	2	1	1
*Staphylococcus hominis*	2	0	2
*Corynebacterium tuberculostearicum*	2	0	2
*Acinetobacter nosocomialis*	1	0	1
*Actinomyces*	1	0	1
*Anaerococcus degeneri*	1	0	1
*Anaerococcus obesiensis*	1	0	1
*Anaerococcus vaginalis*	1	0	1
*Bacteroides coagulans*	1	0	1
*Bulleidia extructa*	1	0	1
*Corynebacterium amycolatum*	1	0	1
*Corynebacterium aurimucosum*	1	0	1
*Corynebacterium coyleae*	1	0	1
*Corynebacterium striatum*	1	0	1
*Corynebacterium simulans*	1	0	1
*Escherichia coli*	1	0	1
*Fenollaria massiliensis*	1	0	1
*Finegoldia magna*	1	0	1
*Gleimia europae*	1	1	0
*Klebisella pneumoniae*	1	0	1
*Klebsiella oxytoca*	1	0	1
*Kylococcus*	1	0	1
*Peptoniphilus grossensis*	1	0	1
*Peptoniphilus harei*	1	0	1
*Peptoniphilus lacrimalis*	1	0	1
*Peptostreptococcus niger*	1	0	1
*Porphyromonas somerae*	1	0	1
*Staphylococcus saccharolyticus*	1	1	0
*Staphylococcus warneri*	1	1	0
*Schaalia funkei*	1	1	0
*Schaalia turcensis*	1	0	1
*Solobacterium moorei*	1	0	1
*Group C streptococcus*	1	0	1
*Peptostreptococcus anaerobius*	1	1	0
Total	120	28	92

**Table 2:** Bacterial strains isolated from deep HS lesions.

Bacterial Strains Isolated from Deep HS Lesions	Total	Female Patients	Male Patients
*Staphylococcus epidermidis*	11	1	10
*Staphylococcus aureus*	7	0	7
*Corynebacterium tuberculostearicum*	6	0	6
*Fenollaria massiliensis*	3	0	3
*Staphylococcus lugdunensis*	3	3	0
*Actinomyces turicensis*	2	0	2
*Actinotignum schaalii*	2	2	0
*Corynebacterium amycolatum*	2	0	2
*Corynebacterium striatum*	2	0	2
*Campylobacter ureolyticus*	2	1	1
*Dialister micraerophilus*	2	0	2
*Fusobacterium gonidiaformans*	2	0	2
*Klebsiella pneumoniae*	2	0	2
*Proteus mirabilis*	2	0	2
*Propionibacterium* spp.	2	1	1
*Coagulase negative staphylococci*	2	1	1
*Staphylococcus haemolyticus*	2	0	2
*Streptococcus constellatus*	2	0	2
*Actinobaculum massiliense*	1	1	0
*Actinomyces europaeus*	1	0	1
*Anaerococcus vaginalis*	1	0	1
*Bacteroides ovatus*	1	0	1
*Cutibacterium acnes*	1	0	1
*Dialister micraerophilus*	1	1	0
*Enterococcus faecalis*	1	1	0
*Fannyhessea vaginae*	1	0	1
*Lactobacillus paracasei*	1	1	0
*Mobiluncus curtisii*	1	0	1
*Parvimonas micra*	1	0	1
*Peptococcus niger*	1	0	1
*Peptoniphilus indolicus*	1	0	1
*Peptoniphilus lacrimalis*	1	0	1
*Peptostreptococcus anaerobius*	1	0	1
*Porphyromonas asaccharolytica*	1	0	1
*Porphyromonas somerae*	1	1	0
*Prevotella disiens*	1	1	0
*Staphylococcus hominis*	1	0	1
*Staphylococcus warneri*	1	0	1
*Slackia exigua*	1	1	0
*Streptococcus agalactiae*	1	0	1
*Streptococcus anginosus*	1	0	1
*Finegoldia magna*	2	2	0
Total	81	18	63

**Table 3:** Antibiotic resistance profile of bacterial strains isolated from HS lesions.

Bacterial Strain	Antibiotic Resistance
*Actinotignum schaalii*	Metronidazole
*Enterococcus faecalis*	Doxycycline
*Escherichia coli*	Ampicillin, Levofloxacin, Co-trimoxazole
*Finegoldia magna*	Clindamycin
*Klebsiella pneumoniae*	Co-trimoxazole
*Propionibacterium* spp.	Metronidazole
*Proteus mirabilis*	Gentamicin
*Staphylococcus aureus*	Penicillin
*Staphylococcus aureus*	Penicillin
*Staphylococcus aureus-MRSA*	Oxacillin, Erythromycin, Clindamycin
*Staphylococcus aureus*	Oxacillin, Erythromycin, Clindamycin
*Staphylococcus aureus-MRSA, MLSBi*	Penicillin, Oxacillin, Erythromycin, Clindamycin, Tetracycline
*Coagulase negative staphylococcus*	Penicillin, Erythromycin, Clindamycin
*Coagulase negative staphylococcus*	Penicillin, Erythromycin, Clindamycin, Tetracycline
*Coagulase negative staphylococcus*	Penicillin, Oxacillin, Tetracycline
*Staphylococcus lugdunensis*	Penicillin, Erythromycin, Clindamycin
*Staphylococcus lugdunensis*	Penicillin
*Group B streptococcus*	Erythromycin, Clindamicyne, Doxycycline

## Data Availability

The datasets generated and analyzed during the current study are available from the corresponding author upon reasonable request.

## Abbreviations

The following abbreviations are used in this manuscript:

HS	Hidradenitis suppurativa
BMI	Body mass index
